# Distribution of Polycyclic Aromatic Hydrocarbons and Pesticides in Danjiangkou Reservoir and Evaluation of Ecological Risk

**DOI:** 10.3390/toxics12120859

**Published:** 2024-11-27

**Authors:** Ruiwen Li, Hao Pang, Yemin Guo, Xuan Zhou, Kaiyu Fu, Taotao Zhang, Jian Han, Lihua Yang, Bingsheng Zhou, Si Zhou

**Affiliations:** 1 Ecology and Environment Monitoring and Scientific Research Center, Ecology and Environment Administration of Yangtze River Basin, Ministry of Ecology and Environment, Wuhan 430010, China; liruiwen@cjjg.mee.gov.cn (R.L.); gym601027@163.com (Y.G.); 2 School of Chemistry and Environmental Engineering, Wuhan Institute of Technology, Wuhan 430074, China; 22209010140@stu.wit.edu.cn (H.P.); hbzhoux@sina.com (X.Z.); 3 Key Laboratory of Breeding Biotechnology and Sustainable Aquaculture, Institute of Hydrobiology, Chinese Academy of Sciences, Wuhan 430072, China; fuky@ihb.ac.cn (K.F.); hanjian@ihb.ac.cn (J.H.); bszhou@ihb.ac.cn (B.Z.); 4 School of Laboratory Medicine, Hubei University of Chinese Medicine, Wuhan 430065, China; vip2564@foxmail.com; 5 Guizhou Institute of Environmental Science and Designing, Guiyang 550081, China

**Keywords:** Danjiangkou Reservoir, risk assessment, polycyclic aromatic hydrocarbons, organophosphorus pesticides

## Abstract

The Danjiangkou Reservoir is the largest artificial freshwater lake in Asia. This study investigated the spatiotemporal distribution of pesticides and polycyclic aromatic hydrocarbons (PAHs) in the Danjiangkou Reservoir to assess the ecological and human health risks associated with these pollutants. Twenty-three sampling sites in the Danjiangkou Reservoir each collected 23 surface water samples and 23 sediment samples. These samples were analyzed using gas chromatography–mass spectrometry (GC–MS), combined with risk quotient methods and health risk assessment models. The results indicated that the total concentration of PAHs (ΣPAHs) in the surface water ranged from 64.64 to 868.23 ng/L (average 217.97 ± 184.97 ng/L), and they primarily consisted of low molecular weight PAHs, with the compounds with the highest concentrations being naphthalene (10.43–116.97 ng/L), fluorene (22.74–87.61 ng/L), and phenanthrene (26.54–162.86 ng/L). The total concentration of pesticides in the surface water varied between 2.62 and 72.89 ng/L (average 22.99 ± 18.27 ng/L). In the sediment samples, the ΣPAH concentration ranged from 0.01 to 2.93 ng/g (average 0.69 ± 0.94 ng/g), and these predominantly consisted of high molecular weight PAHs, while pesticide concentrations ranged from non-detectable (nd) to 28.46 ng/g (average 7.99 ± 8.53 ng/g), with higher concentrations of malathion (0.62–9.16 ng/g) and chlorpyrifos (10.01–21.38 ng/g). Through risk assessment, it was found that although the risks posed by PAHs and pesticides to human health are very low, the ecological risk assessment indicated that certain PAHs (such as phenanthrene) and organophosphate pesticides (such as malathion and chlorpyrifos) may pose potential threats to aquatic organisms.

## 1. Introduction

The Danjiangkou Reservoir, which spans the provinces of Henan and Hubei, holds a distinctive position as the largest artificial freshwater lake in Asia and serves as the confluence of the Han and Dan Rivers. It plays a crucial role in the Middle Route Project of China’s South-to-North Water Diversion initiative, annually supplying the substantial amount of 95 billion cubic meters of clean water to 14 major cities in northern China [1]. The land use in the vicinity of the reservoir encompasses forest land (consisting of natural forests and orchards), agricultural land (which includes dry land and paddy fields), and wetlands, as well as industrial areas. Consequently, the reservoir faces pollution threats from various human activities within its watershed, including industrial discharge, pesticide and fertilizer applications, and livestock manure [2]. Considering its strategic importance in both ecological and socio-economic development, the occurrence of organic pollutants in the aquatic environments of the Danjiangkou Reservoir and their potential harm to aquatic ecosystems and human health have received increasing attention [3,4]. Although previous studies mainly focused on water quality parameters related to eutrophication [5], occurrences of various of organic pollutants including perfluorinated substances [6], antibiotics, and microplastics have been recently detected [1,7]. According to existing studies, Polychlorinated Biphenyls (PCBs), Polybrominated Diphenyl Ethers (PBDEs), and pharmaceuticals and personal care products (PPCPs) have been detected in the Danjiangkou Reservoir. Among these, the concentration of PPCPs is relatively low, while PBDEs pose a significant risk to fish [8,9,10]. Habitat surveys indicate that agricultural activities are frequent near the Danjiangkou Reservoir, leading to the unavoidable use of pesticides and fuel combustion. Although industrial production and healthcare facilities are located relatively far from the reservoir, research on pollutants related to the Danjiangkou Reservoir is still relatively scarce. Current studies are insufficient to support the transition of water environment management goals from merely maintaining water quality standards to protecting ecological and health safety. On the one hand, as China is a leading agricultural nation, the use of pesticides is an important source of water pollution [11,12]. Since some traditional organochlorines (OCPs) have been prohibited in agriculture, more products have been introduced into the market, including organophosphates (OPPs) and pyrethroids. Although OPPs and pyrethroids degrade more readily than organochlorines, they may exhibit toxicity to aquatic organisms such as fish, mollusks, and arthropods, and even pose a potential threat to human health through residues in food [12] such as organophosphates pyrethroids, with some classified as global endocrine disruptors [12]. A pesticide risk assessment of Lake Taihu as well as the Jiulong River in South China found that pesticides pose a risk to the ecosystem and to people [12,13,14]. On the other hand, polycyclic aromatic hydrocarbons (PAHs) are a group of persistent organic pollutants (POPs) with two or more benzene rings, which primarily originate from industrial activities such as fossil fuel, petroleum development, and oil transportation, as well as some natural resources [15,16,17]. Polycyclic aromatic hydrocarbons (PAHs) are widely present in ecological environments [18,19]. Studies that have assessed the risk of PAHs in the Yellow River Delta and along the coast of Bohai Bay have found that PAHs pose ecological risks and harm to aquatic organisms [20,21]. Moreover, When PAHs in drinking water reach a certain concentration, they may pose a risk to human health [22], reminding us to pay attention to these pollutants.

Therefore, given the limited research on the different types of pesticides and polycyclic aromatic hydrocarbons in the region, an investigation was conducted to fill the data gap regarding these pollutants in surface water and sediment samples from the Danjiangkou Reservoir. The main objectives of this study were (1) to analyze the temporal and spatial distribution trends of polycyclic aromatic hydrocarbons and pesticides in the aquatic environment of the Danjiangkou Reservoir, and (2) to assess the ecological and human health risks of contaminants in surface water and sediments in the study area.

## 2. Materials and Methods

### 2.1. Sample Collection and Pretreatment

**Figure 1:**
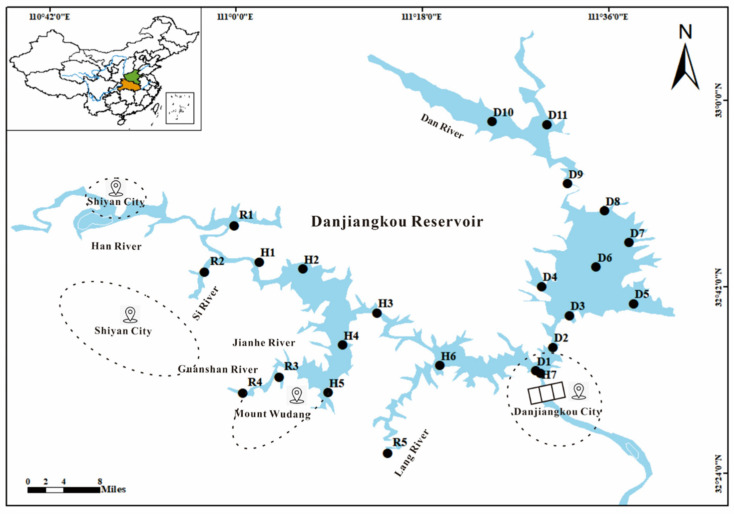
A map showing the sampling sites in the Danjiangkou Reservoir in Hubei Province.

A total of 23 surface water samples and 23 sediment samples were collected from the Danjiangkou Reservoir in August 2020 (Figure 1), after which the surface water samples were analyzed for 10 PAHs and 11 pesticides, and the sediment samples were analyzed for 3 PAHs and 16 pesticides. D1–D11 are sites in the Dan Reservoir, H1–H7 are in the Han Reservoir. R1 is the Han River, R2 is the Si River, R3 is the Jian River, R4 is the Guanshan River, and R5 is the Langhe River in the tributaries of the Danjiangkou Reservoir.

Specifically, surface water samples were collected using 1-L amber glass bottles, with one liter collected per sample, and stored at −4 °C. Within 48 h, the water samples underwent filtration through 3 μm, 0.45 μm, and 0.22 μm microporous filter membranes. After filtration, 50 mL of methanol was added to the samples, which were then stored at 4 °C and the enrichment process was completed within two hours. Enrichment of the water samples was achieved through solid phase extraction (SPE), following established methods [23,24,25].

Sediment samples (200 to 300 g) were collected using a Petersen mud collector, cleared of weeds and debris, placed in washed polythene self-sealing bags, sealed tightly, and stored at −20 °C. The cryopreserved sediment was subsequently dried, homogenized, and sieved through a 200-mesh sieve before being stored again at −20 °C. For extraction, 10 g of the sediment sample was weighed and transferred to the Q-Cup of a fully automated pressurized fluid extraction system. The extraction was conducted at 120 °C using 25 mL of a 1:1 acetone/n-hexane mixture (comprising 20 mL of the upper layer and 5 mL of the lower layer) and 10 mL of rinsing solvent (also 1:1 acetone/n-hexane), with a dwell time of 3 min. Following this, the sample was cleaned twice with 20 mL of dichloromethane. After extraction, we carefully removed the receiver tube and placed the extract on a nitrogen blower. We used a gentle nitrogen flow rate to concentrate the extract down to 1 mL. Purification involved passing the extract through a graphitized carbon black solid-phase extraction column with an aminopropyl-bonded silica gel solid-phase extraction column in series below, both pre-drenched with 4 mL of a toluene-acetonitrile mixture. One gram of anhydrous sodium sulfate was added to the column. The sample was then loaded onto the clean-up column and drenched with 6 mL of the toluene-acetonitrile mixture. The collected drenched solution was concentrated nearly to dryness using a nitrogen blower at a moderate nitrogen flow rate. The solvent was then exchanged for dichloromethane by adding 10 μL of an internal standard solution at a concentration of 400 ppb. The final volume was adjusted to 100 μL for analysis. For further details, please refer to the published article by Black et al. (2023) [26].

### 2.2. Instrumental Analysis

The determination of pesticides in the extracts was conducted using gas chromatography–mass spectrometry (GC–MS) (6890N-5975, Agilent, Santa Clara, CA, USA) with a DB-5 ms (30 m) column (Agilent, Santa Clara, CA, USA). The GC conditions involved an initial hold at 40 °C for 1 min, followed by a ramp to 130 °C at 30 °C/min, another ramp to 250 °C at 5 °C/min, a further ramp to 280 °C at 10 °C/min, and a final hold at 280 °C for 8 min. The inlet temperature was set to 270 °C, with a shunt injection method (10:1 shunt ratio) using a 1.0 μL injection volume. Helium served as the carrier gas at a flow rate of 1.4 mL/min. For MS conditions, an EI source was employed with a temperature of 230 °C for the ion source and 280 °C for the interface. The ionization energy was set to 70 eV, and the scanning mode used for quantification was the ion scanning mode (SIM), with the full scanning mode (SCAN) used for qualitative reference. A solvent delay time of 4.3 min was applied. A total of 21 contaminants were detected in the surface water, primarily divided into three categories: 10 polycyclic aromatic hydrocarbons (PAHs), 8 organophosphates, and 3 organochlorines. In the sediments, 19 contaminants were detected, categorized into four groups: 3 polycyclic aromatic hydrocarbons, 3 pyrethroids, 8 organophosphate esters, and 5 organochlorines.

### 2.3. Quality Assurance/Quality Control

The QA/QC method mainly includes spiked blank, method blank, matrix spiked parallel, and sample parallel samples, and the whole experimental process refers to the QA/QC quality control system of the USEPA TO-13A method [27]. One liter each of ultrapure water and blank filter membrane were used as the field blank and experimental process blank samples, respectively, and the field blank, process blank, and collected samples were subjected to the same extraction, purification, and analytical detection processes to evaluate possible contamination during the whole experimental process. The spiked recoveries of pesticides in the water and sediment samples ranged from 60% to 140%. The method detection limits and method quantification limits are shown in Table S2.

### 2.4. Ecological Risk Assessment

Ecological risk assessments of pesticides in the Danjiangkou Reservoir were performed using the Hazard Quotient (HQ) method [28,29,30]. Data regarding the toxicity of pesticides to aquatic organisms were collected from the ECOTOX database (https://cfpub.epa.gov/ecotox/ (accessed on 16 October 2023)) and other published research (see Tables S4 and S5 for details). The HQ was calculated as follows:

(1) $${HQ}_{water}={EC}_{water}/{PNEC}_{water}$$

(2) $${PNEC}_{water}={L(E)C}_{50}/AF$$

where EC_water_ is the contaminant concentrations in water, ng/L. PNEC_water_ is the predicted no effect concentration of contaminants in water, mg/L. AF is the evaluation factor, which is taken as 1000 according to the EU Water Framework Directive. L(E)C_50_ is the median lethal concentration of the tested organisms. ECOSAR software 2.2 was used to simulate the toxicity data of the pesticides in relation to fish, daphnia, and green algae (Tables S4 and S5). When HQ_water_ < 0.01 and there is no detectable concentration, the ecological risk is insignificant. When 0.01 < HQ_water_ < 0.1, the ecological risk is low. When 0.1 < HQ_water_ < 1, the ecological risk is medium. When HQ_water_ ≥ 1, the ecological risk is high.

According to EU technical guidelines on environmental risk assessment, PNEC_sed_ (ng/g) was calculated by the equilibrium partitioning method.

(3) $${\mathrm{PNEC}}_{\mathrm{sediment}}=\frac{K_{\mathrm{susp}-\mathrm{water}}\times {1000\times \mathrm{PNEC}}_{\mathrm{water}}}{{\mathrm{RHO}}_{\mathrm{susp}}}$$

(4) $$K_{\mathrm{susp}-\mathrm{water}}=F_{\mathrm{water}-\mathrm{susp}}{+F}_{\mathrm{solid}-\mathrm{susp}}\times F_{\mathrm{oc}-\mathrm{susp}}\times \frac{K_{\mathrm{oc}}}{1000}\times {\mathrm{RHO}}_{\mathrm{solid}}$$

where *K*_susp-water_ is the suspended matter-water partitioning coefficient (m^3^/m^3^). RHO_susp_ is the suspended solids capacity (1150 kg/m^3^). RHO_solid_ is the density of the solid phase (2500 kg/m^3^). F_water-susp_ is the volume ratio of water in suspended matter (0.9 m^3^/m^3^). F_solid-susp_ is the volume ratio of solids in suspended matter (0.1 m^3^/m^3^). F_oc-susp_ is the mass fraction of organic carbon in suspended matter (0.1 kg/kg). *K*_oc_ represents the absorption constants of organic compounds (L/kg), which can be obtained by EPI suit v4.11.

(5) $${HQ}_{sed}={EC}_{sed}/{PNEC}_{sed}$$

HQ is used to assess the ecological risk of contaminants in samples from the Danjiangkou Reservoir. EC_sed_ is the concentration of the contaminant in the sediment (ng/g). For compounds with log K_ow_ values less than 5, the quotient HQ_sed_ is compared to 1 (threshold). For compounds with log K_ow_ values greater than 5, the HQ_sed_ is compared to 10. This is because contaminants with log K_ow_ values greater than 5 have a higher capacity for sediment adsorption and are less harmful to benthic organisms, so the evaluation criteria can be relaxed [28].

### 2.5. Human Health Risk Assessment

To estimate human health risks associated with exposure to PAHs and pesticides, the U.S. Environmental Protection Agency’s (EPA) health risk assessment model was employed to quantify both carcinogenic and non-carcinogenic risks. Exposure to these substances in water occurs through dermal contact and ingestion, typically during swimming and drinking. Similarly, exposure to sediments happens via ingestion of soil and dermal contact with it. To represent a worst-case exposure scenario, risk calculations utilized adult body weight values (70 kg). The methodology for integrating toxicity and exposure data in this study adhered to the risk assessment guidelines [31]. Assessments were conducted for risks arising from ingestion of surface water and sediment, as well as dermal contact with both.

$$CDI\left(Water\;Ingestion\right)=\frac{CW\times IR(W)\times EF\times ED}{BW\times AT}$$

CDI (Water Dermal) was calculated with reference to a previous study [32].

$$CDI\left(Water\;Dermal\right)=\frac{(6\times \tau \times \frac{TE}{\pi }{)}^{0.5}\times CW\times k\times SA\times EF\times FE\times ED}{BW\times AT\times f\times 500}$$

where CDI is the daily exposure of the *i*-th contamination (mg/kg·d). CW is the chemical concentration in water (mg/L). IR(W) is the ingestion rate. EF is the exposure frequency. ED is the exposure duration. BW is the body weight. AT is the averaging time. TE is the event duration. f is the intestinal absorption ratio. k is a skin permeability parameter. τ is the lag time for each pollutant in the body. FE is the event frequency. SA is the skin surface area available for contact. ET is the exposure time. CF is the volumetric conversion factor for water.

$$CDI\left(Sediment\;Ingestion\right)=\frac{CS\times IR(S)\times CF\times FI\times EF\times ED}{BW\times AT}$$

$$CDI\left(Sediment\;Dermal\right)=\frac{CS\times SA\times AF\times ABS\times EF\times ED\times CF}{BW\times AT}$$

where CS is the chemical concentration in soil (mg/kg). IR(S) is the ingestion rate (mg/d). CF is the conversion factor. FI is the fraction ingested from contaminated sources. EF is the exposure frequency. ED is the exposure duration. BW is the body weight. AT is the averaging time. SA is the skin surface area available for contact. AF is the soil to skin adherence factor. ABS is the absorption factor.

The non-carcinogenic risk assessment model is as follows [33]:

$$HQ=\frac{CDI}{RfD}$$

$$HI=\sum_{i=1}^{n}{HQ}_{i}$$

where RfD denotes the reference dose (mg/kg·d). HQ is the non-carcinogenic risk index. HI is the total non-carcinogenic risk index. In order to estimate the total health risk from complex pollutants (risk from exposure to different pollutants), the hazard index was applied. The HI was estimated as the sum of the HQs for the individual pollutants. The HI exceeded 1, which means that it presents a potentially adverse human health effect [34].

The carcinogenic risk assessment model is as follows:

$$CR_{i}={CDI}_{i}\times {SF}_{i}$$

$$TCR=\sum_{i=1}^{n}{CR}_{i}$$

where SF is the carcinogenic slope factor (mg/kg·d). R_i_ is the carcinogenic risk index. TCR is the total carcinogenic risk index. For most regulatory programs, a carcinogenic risk value between 10^−6^ and 10^−4^ indicates a potential risk, while a carcinogenic risk value of 10^−6^ or less indicates a safe level, and a carcinogenic risk value greater than 10^−4^ indicates a potentially high health risk.

## 3. Results and Discussion

### 3.1. Occurrence and Spatial Distribution of Contaminants in Surface Water of Danjiangkou Reservoir

**Figure 2:**
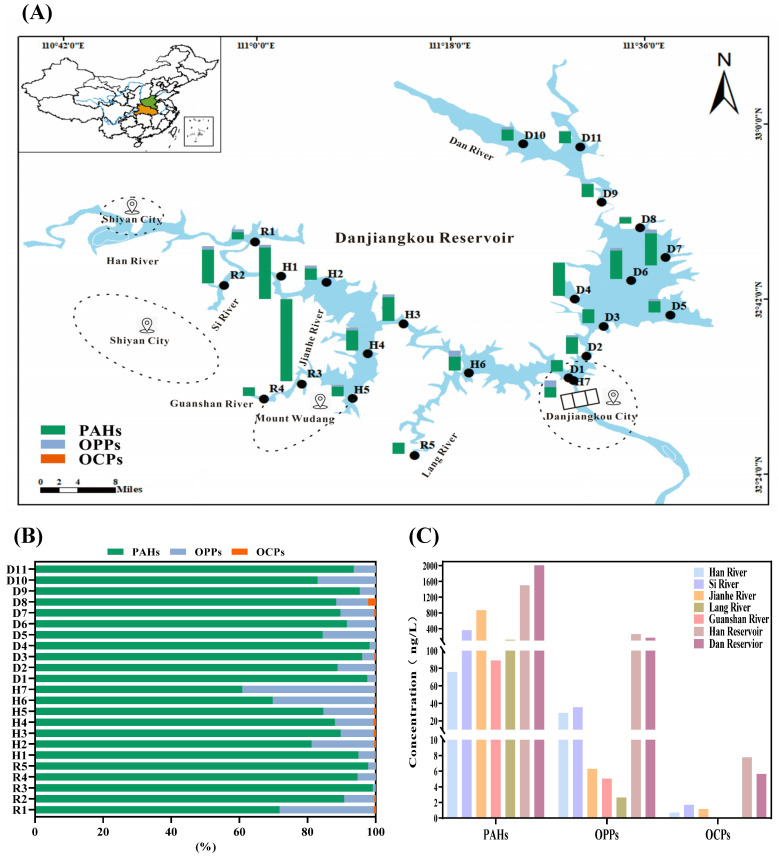
The occurrence of three types of organic pollutants in surface waters of the Danjiangkou Reservoir. (**A**) The spatial distributions of three types of organic pollutants in the Danjiangkou Reservoir. (**B**) The composition of three types of organic pollutants in surface water of the Danjiangkou Reservoir. (**C**) A histogram of the organic pollutant concentrations in different areas of the Danjiangkou Reservoir. PAHs: polycyclic aromatic hydrocarbons; OPPs: organophosphates; OCPs: organochlorines.

In water samples collected from the Danjiangkou Reservoir, a total of 21 pollutants were detected, including 10 PAHs and 11 pesticides (Table 1). As shown in Figure 2, it is evident that throughout the 23 monitoring points, polycyclic aromatic hydrocarbons (PAHs) dominate, with proportions ranging from 60.79% to 99.15%. Organic phosphorus pesticides vary in proportion from 0.72% to 38.90%, while organochlorine pesticides exhibit low detection concentrations across all measuring points, with proportions ranging from 0.00% to 2.22%. These results are consistent with previous conclusions drawn from surface water in the Yongding River basin [35].

Specifically, the total concentration of PAHs (∑PAHs) in the reservoir’s water ranged from 64.64 to 868.23 ng/L, averaging at 217.97 ± 184.97 ng/L. In comparison to other Chinese rivers, such as the Qiantang River (70.3 to 1844.4 ng/L, average: 283.3 ng/L), the Yellow River (144.3 to 2360 ng/L, average: 662 ng/L), and the Daliao River Estuary and adjacent areas (71.12 to 4255.43 ng/L, average: 748.76 ng/L), the ∑PAH concentrations in the Danjiangkou Reservoir were relatively lower [36,37,38], and the concentration of ∑PAHs in the Danjiangkou Reservoir was also low compared to that in foreign countries, such as in the Mahanadi River Basin (13.1–685.4 μg/L) and on the Mediterranean coast (13,156–34,852 ng/g) [39,40]. However, a comparison of the concentrations of specific pollutants revealed that both Nap and Phe were the pollutants with the highest detected concentrations. As shown in Figure 2, the concentration of ∑PAHs varied among the tributary sampling sites, and among all sampling sites, the highest concentration was found at the Guanshan River (R3). This may be attributed to the intensive human activity and fossil fuel combustion in nearby areas [41]. In contrast, the concentration of ∑PAHs presented small fluctuations among the sampling sites in the Han and Dan Reservoirs, resulting in higher average concentrations than some tributary rivers (i.e., Jianhe River and Lang River) with less human activity. The sources of PAHs in the Han and Dan Reservoirs may include the input from tributary rivers as well as ship operations and oil leakage [42]. Notably, PAHs containing 2–3 benzene rings and 4 benzene rings (low molecular weight PAHs, LMW-PAHs) were predominant in surface water from the Danjiangkou Reservoir, with naphthalene as the most predominant. This is similar to findings in the Three Gorges Reservoir [43]. Naphthalene is a major product of diesel and gasoline, possibly originating from incomplete combustion sources, while pyrene is indicative of coal combustion and benzo[a]pyrene is a marker for fuel combustion. Therefore, it can be inferred that the primary sources of PAHs in the study area are petroleum pollution and combustion of petroleum products [44].

The total concentrations of organophosphorus pesticides (∑OPPs) in surface water from the Danjiangkou Reservoir ranged from 2.62 to 71.14 ng/L, averaging at 22.26 ± 18.06 ng/L, which were much lower than those in the Zhujiang Estuary (∑OPPs: 460–43,600 ng/L) and in the water samples of Jiangsu Province (∑OPPs: 63.7–22,463 ng/L) [45], and the concentrations in surface water samples from foreign countries were lower than those of the Tiber River (∑OPPs: 0.40–224.48 ng/L) [46]. Among the detected OPPs, atrazine and dichlorvos had higher detection rates and average concentrations, which is consistent with the findings of previous studies [47]. Spatially, the concentrations of ∑OPPs were higher in the Han Reservoir and Dan Reservoir than in the tributary sampling sites. Additionally, the Han River (R1) and Si River (R2) had higher concentrations of ∑OPPs than other tributaries, possibly due to higher levels of agricultural activity in these locations [48].

Furthermore, the study revealed that the total concentration of organochlorine pesticides (∑OCPs) in surface water within the research area varied from non-detectable levels to 1.75 ng/L, averaging at 0.73 ± 0.66 ng/L. According to previous studies, low levels of organochlorine pesticides (OCPs) were detected in the Xujia River Basin, the Beiluo River, and the Irtysh River, which were similar to those found in the surface water of the Danjiangkou Reservoir, with high detection rates of α-HCH, β-HCH, and γ-HCH [49,50,51]. The lower concentrations of organochlorine pesticides compared with organophosphorus pesticides may be related to the restriction of the production and use of certain OCPs since 1983 and the ratification of the Stockholm Convention on Persistent Organic Pollutants (POPs) [52]. The lower concentrations of ∑OCPs at the tributary sampling sites also indicate limited input from neighboring regions, and the relatively higher concentrations in the Han Reservoir and Dan Reservoir may be related to the extensive historical use and persistence of organochlorine pesticides [3,53,54]. Among the detected OCPs, HCH-alpha had the highest detection frequency and concentration, which may primarily stem from residual organochlorine pesticide usage in the past [55].

Taken together, the total concentrations of detected pollutants ranged from nondetectable levels (ND) to several thousand nanograms per liter (ng/L), aligning with findings from global studies [28,56,57]. Our results also indicate that polycyclic aromatic hydrocarbons (PAHs), specifically Nap, Flu, and Phe, are the primary pollutants in the surface water of the Danjiangkou Reservoir. The Han Reservoir and Dan Reservoir had higher concentrations of all the three types of detected pollutants (PAHs, OPPs, and OCPs) compared to the tributaries, suggesting that the reservoirs may have a sedimentation and aggregation effect on the surrounding input pollutants.

### 3.2. Occurrence and Spatial Distribution of Contaminants in Sediments of Danjiangkou Reservoir

**Figure 3:**
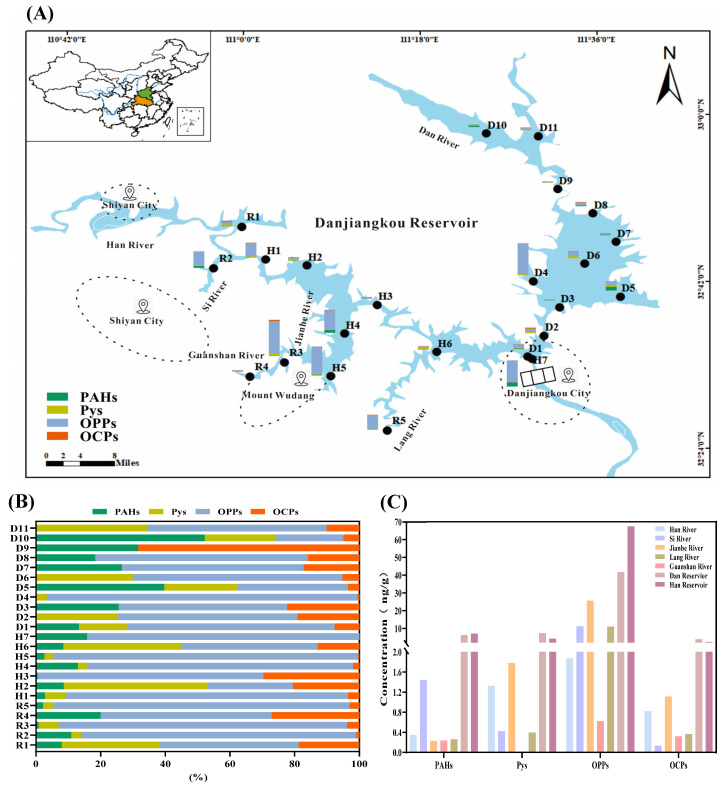
The occurrence of four types of organic pollutants in sediment of the Danjiangkou Reservoir. (**A**) The spatial distributions of four types of organic pollutants in the Danjiangkou Reservoir. (**B**) The composition of four types of organic pollutants in sediment of the Danjiangkou Reservoir. (**C**) A histogram of organic pollutant concentrations in different areas of the Danjiangkou Reservoir. PAHs: polycyclic aromatic hydrocarbons; Pys: pyrethroids; OPPs: organophosphates; OCPs: organochlorines.

In sediment samples collected from the Danjiangkou Reservoir, a total of 19 pollutants were analyzed, including 3 PAHs, 8 organophosphates, and 5 organochlorines (Table 2 and Figure 3). The total concentration of PAHs in the sediment samples ranged from 0.01 to 2.93 ng/g (average: 0.69 ± 0.94 ng/g), significantly lower than the PAH concentrations found in the Yangtze River Wuhan segment (72.4–3995.2 ng/g) and the Yangtze River Delta urban agglomeration (493–15,907 ng/g; average: 2483 ng/g) [58,59]. Furthermore, the PAH concentrations in the Danjiangkou Reservoir sediment were much lower than those in other Chinese reservoirs like the Chahe Reservoir (642.94–1495.96 ng/g) and the Fenhe Reservoir and its basin (539.0–6281.7 ng/g; average: 2214.8 ng/g) [60,61]. The Mahanadi River, India (13.1–685.4 ng/g), and the Tiber River, Italy (4.5–652.2 ng/g), also had much higher concentrations than those measured in sediments from the Danjiangkou Reservoir [62]. These results suggest a relatively minimal pollution level of PAHs in the Danjiangkou Reservoir. In contrast with the above results of the surface water samples, the detection rates of high molecular weight (HMW) PAHs were all above 60%, whereas LMW PAHs were not detected in the sediment samples. This may be because HMW PAHs (4–6 ring PAHs) have stronger oil affinity, lower volatility, and lower water solubility, and are more prone to accumulate in sediments, potentially posing carcinogenic effects in humans [63,64,65].

Unlike the surface water results, organophosphate pesticides (OPPs) were dominant in the sediment at most sampling sites. The total concentration of ∑OPPs in the reservoir sediment ranged from nd-25.57 ng/g (average: 6.93 ± 8.41 ng/g), which is much lower than that reported in the Huai River basin (2951–47,739 ng/g; average: 8041 ng/g) and the Yangtze River basin (3022–12,756 ng/g; average: 5936 ng/g) [45]. Among the detected OPPs, the highest detection frequency of 65.22% was observed for TRANS, indicating its more prevalent use in adjacent areas. Additionally, the occurrence of pyrethroids was detected in the sediment samples from the Danjiangkou Reservoir, with the total concentrations ranging from nd-1.78 ng/g (average: 0.67 ± 0.60 ng/g). These were lower than the concentrations found in sediment samples from urban rivers in Guangzhou in both the dry season (0.25–3113 ng/g; average: 217 ng/g) and the wet season (0.25–4732 ng/g; average: 388 ng/g) [66]. The pyrethroid concentrations in the Danjiangkou Reservoir sediment were also lower than those in sediments from the Liao River basin (2.2–102.5 ng/g; average: 17.3 ng/g) [48]. Furthermore, the total concentrations of organochlorine pesticides (OCPs) in the sediment samples from the Danjiangkou Reservoir (nd-1.11 ng/g, average: 0.39 ± 0.27 ng/g) were comparable to the reported concentrations in sediments from the Dongping Lake in North China and the Chennab River in Pakistan [67,68], but much lower than those detected in the Huai River basin (531–6533 ng/g; average: 1408 ng/g) and the Yangtze River basin (305–2190 ng/g; average: 1077 ng/g) [45]. Among the detected OCPs, beta-hexachlorocyclohexane (β-HCH) had the highest detection rate (exceeding 80%) and dieldrin had the highest concentration (up to 0.98 ng/g). These results indicate relatively low environmental concentrations in the sediment of the Danjiangkou Reservoir.

Taken together, the components of the detected pollutants in the sediment varied from those in the surface water of the Danjiangkou Reservoir. For one thing, the OPPs were dominant at most sampling sites, and the occurrence of pyrethroids was detected in the sediment. For another, HMW PAHs were the primary PAHs in the sediment, and no LMW PAHs were detected. However, the average concentrations of all three types of detected pollutants (PAHs, OPPs, and OCPs) were also higher in the Han Reservoir and Dan Reservoir than the tributaries, which is consistent with the findings in surface water. These results also confirm the speculation that the reservoirs have a sedimentation and aggregation effect on the surrounding input pollutants.

### 3.3. Ecological Risk Assessment of Danjiangkou Reservoir

During this study, we conducted an ecological risk assessment of the Danjiangkou Reservoir by examining the potential risks for three trophic levels: green algae, water fleas, and fish. The hazard quotient (HQ) was employed as an effective method to evaluate risks based on measured environmental concentrations [28]. An HQ value greater than 1 indicates high ecological risk, between 0.1 and 1 signifies moderate risk, less than 0.1 indicates low risk, and values below 0.01 or undetectable pollutant concentrations are considered insignificant.

**Figure 4:**
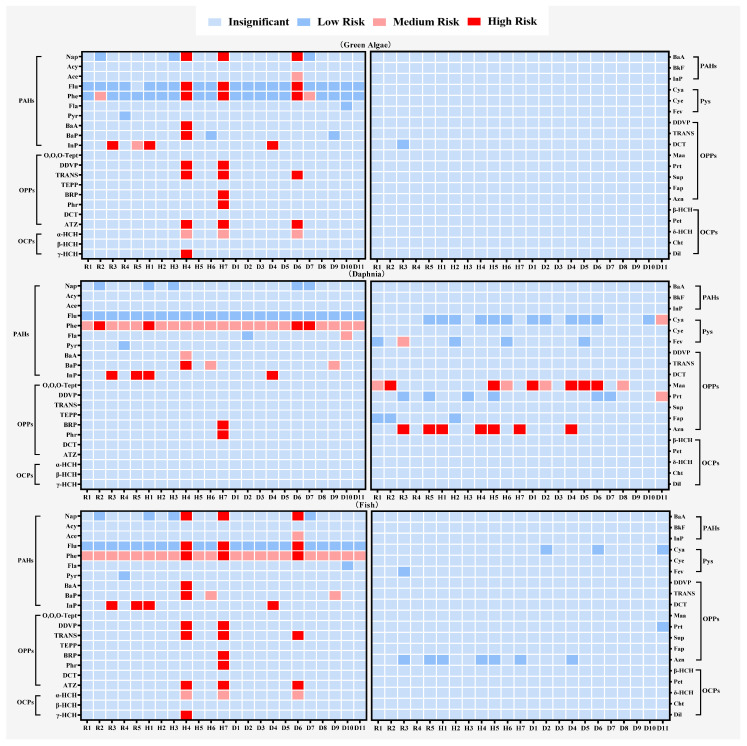
A heatmap of the risk quotients of the organic pollutants detected at each sampling point for the three most sensitive species. Hazard quotients (HQs) are based on the concentrations of organic pollutants measured in surface water and sediment at each sampling point in the Danjiangkou Reservoir. Nap: Naphthalene; Acy: Acenaphthylene; Ace: Acenaphthene; Flu: Fluorene; Phe: Phenanthrene; Fla: Fluoranthene; Pyr: Pyrene; BaA: Benz[a]anthracene; BaP: Benzo[a]pyrene; InP: Indeno[1,2,3-cd]pyrene; O,O,O-Tept: O,O,O-triethyl-Phosphorothioate; DDVP: Dichlorvos; TRANS: E-Mevinphos; TEPP: Tetraethyl pyrophosphate; BRP: Naled; Phr: Phorate; DCT: Simazine; ATZ: Atrazine; BkF: Benzo[k]fluoranthene; Cya: Cyhalothrin (Lambda); Cye: Cypermethrin I; Fev: Fenvalerate I; DCT: Simazine; Maa: Malathion; Prt: Prothiofos; Sup: Sulprofos; Fap: Famphur; Azn: Azinphos-methyl; Pet: Pentachloronitrobenzene; Cht: Chlordane-trans; Dil: Dieldrin.

Among the contaminants detected in surface water from the Danjiangkou Reservoir, naphthalene, fluorene, and phenanthrene may pose high risks to green algae and fish at the H4, H7, and D6 locations, and Phe may pose medium to high risks to water fleas and fish at all sampling sites (Figure 4). At H4, BaA and BaP were found to pose high risks to green algae and fish. InP posed medium to high risks to all three species at the R2, R5, H1, and D4 locations. Additionally, organophosphate pesticides such as DDVP, TRANS, BRP, Phr, and ATZ posed high risks to green algae and fish at certain points. BRP and Phr posed high risks to water fleas at H7. Among the three organochlorine pesticides, α-HCH showed medium risks for green algae and fish at three identical points, while Lindane (γ-HCH) posed high risks for green algae and fish at the same location. Taken together, PAHs such as phenanthrene may pose higher risks to water fleas and fish compared to other detected contaminants, making them priority pollutants that require further attention. In addition, aquatic organisms living around sampling sites H4, H7, and D6 may encounter moderate to high risks due to the presence of various pollutants, necessitating closer scrutiny of emissions from nearby pollution sources.

To evaluate the ecological risks posed by contaminants detected in the sediments from the Danjiangkou Reservoir, based on the guidelines recommended by the European Union, the equilibrium partitioning method was utilized to calculate the predicted non-effect concentration [28]. As shown in Figure 4, neither PAHs nor organochlorine pesticides in sediment would pose potential risks to the three categories of organisms, whereas Cya, Fev, and Azn may pose low risks to fish at specific monitoring sites. However, low to medium risks for water fleas were observed in three pyrethroid pesticides at certain sites (R5 to D11). In contrast, the organophosphate pesticides Maa and Azn may pose a high level of risk to water fleas at certain sites (Maa: R2, H5, D1, D4, D5, and D6; Azn: R3, R5, H1, H4, H5, H7, and D4). This may be related to their higher concentrations in sediments as abovementioned in the chemical analysis results.

Overall, our results suggest that water fleas are more sensitive to the contaminants in sediment of the reservoir, especially Maa and Azn. These observations also indicate that organophosphate pesticides might be the primary risk sources in the sediment, which should be considered a priority.

### 3.4. Human Health Risk Assessment of Danjiangkou Reservoir

The non-carcinogenic and carcinogenic risks were calculated following USEPA protocols to evaluate whether the detected contaminants in surface water could cause any potential threat to human health [69]. Table 3, Tables S8 and S10 list the HIs obtained by the non-carcinogenic risk evaluation of the contaminants in surface water and sediment at different sampling sites in the Danjiangkou Reservoir, and all the calculated HIs obtained were less than 1, which indicates that these contaminants may not have negative health effects [70]. Table 4, Tables S9 and S11 show the TCRs obtained from the carcinogenic risk evaluation of the pollutants in surface water and sediments in the study area, and the calculated values were all below 10^−6^, indicating that these contaminants may not pose a carcinogenic risk.

It should be noted that the risk assessment in this study has certain limitations: (1) criteria derivation uncertainty. During the process of deriving PNECsediment, this study adopted the equilibrium distribution model based on some theoretical assumptions, and the differences between these assumptions and the real situation will certainly bring some uncertainty to the evaluation results; (2) risk assessment uncertainty. During the risk assessment based on the HQ method, the HQ is constructed based on the small amount of existing sediment toxicity data. A small amount of sediment toxicity data may cause uncertainty in the evaluation results. However, this study conducted risk assessments based on the detection concentrations of field samples from the water source area. In fact, the water still needs to undergo purification in a water treatment plant before it can be supplied for human consumption. Therefore, we can consider the Danjiangkou Reservoir as a safe water source.

## 4. Conclusions

This study primarily monitored the surface water and sediments of the Danjiangkou Reservoir, revealing the presence of various organic pollutants in the aquatic environment, including polycyclic aromatic hydrocarbons (PAHs) and pesticides. The concentration ranges detected in the surface water samples were as follows: ∑PAHs (64.64–868.23 ng/L, average concentration 217.97 ± 184.97 ng/L), ∑OPPs (2.62–71.14 ng/L, average concentration 22.26 ± 18.06 ng/L), and ∑OCPs (nd-1.75 ng/L, average concentration 0.73 ± 0.66 ng/L). In the sediments, the concentration ranges were as follows: ∑PAHs (0.01–2.93 ng/g, average concentration 0.69 ± 0.94 ng/g), ∑OPPs (nd-25.57 ng/g, average concentration 6.93 ± 8.41 ng/g), ∑Pys (nd-1.78 ng/g, average concentration 0.67 ± 0.60 ng/g), and ∑OCPs (nd-1.11 ng/g, average concentration 0.39 ± 0.27 ng/g). Although the concentrations of these pollutants are relatively low and show no significant differences compared to reports from other national and international river basins, assessments using the health risk evaluation model of the U.S. Environmental Protection Agency (EPA) indicate that they currently pose minimal risks to human health. However, ecological risk assessments conducted using the hazard quotients reveal potential risks posed by these pollutants to aquatic organisms, especially certain polycyclic aromatic hydrocarbons (such as phenanthrene) and organophosphate pesticides (such as malathion and chlorpyrifos). Therefore, continued monitoring and risk assessment are necessary to ensure the protection of aquatic ecosystems in the Danjiangkou Reservoir.

## Figures and Tables

**Table 1:** The detection results of PAHs, organophosphates, and organochlorine pesticides and their isomers in water samples from the Danjiangkou Reservoir.

Targets	Analyte	Abbreviation	Water (n = 29) ng/L			
			DF (%)	Min	Max	Mean
PAHs	Naphthalene	Nap	100.0	10.43	116.97	41.06
	Acenaphthylene	Acy	-	ND	ND	-
	Acenaphthene	Ace	4.4	ND	0.22	0.11
	Fluorene	Flu	100.0	22.74	87.61	42.45
	Phenanthrene	Phe	100.0	26.54	162.86	71.43
	Fluoranthene	Fla	8.7	ND	7.56	5.64
	Pyrene	Pyr	4.4	ND	12.04	12.04
	Benz[a]anthracene	BaA	4.4	ND	13.12	13.12
	Benzo[a]pyrene	BaP	13.0	ND	66.40	28.61
	Indeno[1,2,3-cd]pyrene	InP	17.4	ND	750.42	331.85
OPPs	O,O,O-triethyl-Phosphorothioate	O,O,O-Tept	-	ND	ND	-
	Dichlorvos	DDVP	78.3	ND	3.15	2.57
	E-Mevinphos	TRANS	60.9	ND	9.56	9.23
	Tetraethyl pyrophosphate	TEPP	4.4	ND	38.74	38.74
	Naled	BRP	4.4	ND	40.00	40.00
	Phorate	Phr	4.4	ND	2.39	2.39
	Simazine	DCT	-	ND	ND	-
	Atrazine	ATZ	91.3	ND	29.03	12.29
OCPs	α-HCH	α-HCH	95.7	ND	40.96	0.61
	β-HCH	β-HCH	43.5	ND	97.73	1.09
	γ-HCH	γ-HCH	65.2	ND	0.72	1.11

DF: Detection frequency. ND: Means a concentration below the method detection limit.

**Table 2:** The detection results of PAHs, pyrethroids, organophosphates, and organochlorine pesticides and their isomers in sediments from the Danjiangkou Reservoir.

Targets	Analyte	Abbreviation	Sediment (n = 23) ng/g			
			DF (%)	Min	Max	Mean
PAHs	Benz[a]anthracene	BaA	100.0	0.00313	0.01	0.01
	Benzo[k]fluoranthene	BkF	60.9	ND	2.87	0.77
	Indeno[1,2,3-cd]pyrene	InP	69.6	ND	0.75	0.31
Pys	Cyhalothrin (Lambda)	Cya	56.5	ND	1.73	0.65
	Cypermethrin I	Cye	26.1	ND	0.72	0.47
	Fenvalerate I	Fev	21.7	ND	1.78	0.82
OPPs	Dichlorvos	DDVP	17.4	ND	0.85	0.31
	E-Mevinphos	TRANS	65.2	ND	0.72	0.54
	Simazine	DCT	8.7	ND	2.23	1.38
	Malathion	Maa	43.5	ND	9.16	2.61
	Prothiofos	Prt	43.5	ND	1.46	0.66
	Sulprofos	Sup	-	ND	ND	-
	Famphur	Fap	13.0	ND	1.53	1.18
	Azinphos-methyl	Azn	30.4	ND	21.38	15.87
OCPs	β-HCH	β-HCH	87.0	ND	0.87	0.26
	Pentachloronitrobenzene	Pet	52.2	ND	0.13	0.10
	δ-HCH	δ-HCH	30.4	ND	0.21	0.17
	Chlordane-trans	Cht	13.0	ND	0.16	0.10
	Dieldrin	Dil	4.4	ND	0.98	0.98

DF: Detection frequency. ND: Means a concentration below the method detection limit.

**Table 3:** Health risks of typical pollutants in surface water of Danjiangkou Reservoir.

Site	Non-Carcinogenic Risks of Surface Water						Carcinogenic Risks of Surface Water		
	Naphthalene	Fluorene	Phenanthrene	Dichlorvos	Atrazine	α-HCH	Naphthalene	Dichlorvos	α-HCH
R1	3.02 × 10^−5^	1.79 × 10^−5^	2.82 × 10^−5^	1.37 × 10^−4^	1.44 × 10^−5^	2.41 × 10^−6^	7.25 × 10^−8^	1.98 × 10^−8^	1.22 × 10^−7^
R2	1.67 × 10^−4^	5.74 × 10^−5^	1.55 × 10^−4^	0	2.12 × 10^−5^	1.99 × 10^−6^	4.01 × 10^−7^	0	1.00 × 10^−7^
R3	2.66 × 10^−5^	3.39 × 10^−5^	4.92 × 10^−5^	1.44 × 10^−4^	3.06 × 10^−6^	0	6.38 × 10^−8^	2.09 × 10^−8^	0
R4	3.27 × 10^−5^	1.86 × 10^−5^	2.67 × 10^−5^	1.56 × 10^−4^	1.88 × 10^−6^	0	7.84 × 10^−8^	2.26 × 10^−8^	0
R5	2.66 × 10^−5^	1.62 × 10^−5^	2.53 × 10^−5^	1.50 × 10^−4^	0	0	6.39 × 10^−8^	2.17 × 10^−8^	0
H1	9.64 × 10^−5^	4.18 × 10^−5^	1.28 × 10^−4^	1.21 × 10^−4^	1.34 × 10^−5^	2.03 × 10^−6^	2.31 × 10^−7^	1.76 × 10^−8^	1.02 × 10^−7^
H2	4.40 × 10^−5^	2.01 × 10^−5^	6.04 × 10^−5^	1.40 × 10^−4^	1.31 × 10^−5^	2.50 × 10^−6^	1.06 × 10^−7^	2.03 × 10^−8^	1.26 × 10^−7^
H3	1.25 × 10^−4^	4.57 × 10^−5^	9.47 × 10^−5^	0	1.47 × 10^−5^	2.47 × 10^−6^	3.00 × 10^−7^	0	1.25 × 10^−7^
H4	4.40 × 10^−5^	2.01 × 10^−5^	7.30 × 10^−5^	1.43 × 10^−4^	1.30 × 10^−5^	2.25 × 10^−6^	1.06 × 10^−7^	2.07 × 10^−8^	1.13 × 10^−7^
H5	3.13 × 10^−5^	1.89 × 10^−5^	5.31 × 10^−5^	1.25 × 10^−4^	5.71 × 10^−6^	2.38 × 10^−6^	7.51 × 10^−8^	1.81 × 10^−8^	1.20 × 10^−7^
H6	4.72 × 10^−5^	2.51 × 10^−5^	6.31 × 10^−5^	1.58 × 10^−4^	9.61 × 10^−6^	2.56 × 10^−6^	1.13 × 10^−7^	2.29 × 10^−8^	1.29 × 10^−7^
H7	4.99 × 10^−5^	2.47 × 10^−5^	3.97 × 10^−5^	1.34 × 10^−4^	1.41 × 10^−5^	2.04 × 10^−6^	1.20 × 10^−7^	1.95 × 10^−8^	1.03 × 10^−7^
D1	3.20 × 10^−5^	3.05 × 10^−5^	5.49 × 10^−5^	1.80 × 10^−4^	0	0	7.69 × 10^−8^	2.61 × 10^−8^	0
D2	5.24 × 10^−5^	3.60 × 10^−5^	8.13 × 10^−5^	0	1.02 × 10^−5^	2.06 × 10^−6^	1.26 × 10^−7^	0	1.04 × 10^−7^
D3	2.82 × 10^−5^	3.76 × 10^−5^	6.92 × 10^−5^	1.54 × 10^−4^	2.24 × 10^−6^	2.23 × 10^−6^	6.76 × 10^−8^	2.23 × 10^−8^	1.12 × 10^−7^
D4	4.01 × 10^−5^	2.75 × 10^−5^	3.77 × 10^−5^	1.47 × 10^−4^	2.87 × 10^−6^	1.90 × 10^−6^	9.63 × 10^−8^	2.14 × 10^−8^	9.56 × 10^−8^
D5	4.25 × 10^−5^	2.80 × 10^−5^	5.00 × 10^−5^	0	1.07 × 10^−5^	0	1.02 × 10^−7^	0	0
D6	1.49 × 10^−4^	4.72 × 10^−5^	1.26 × 10^−4^	0	1.50 × 10^−5^	2.00 × 10^−6^	3.59 × 10^−7^	0	1.01 × 10^−7^
D7	1.51 × 10^−4^	6.26 × 10^−5^	1.45 × 10^−4^	0	2.37 × 10^−5^	2.20 × 10^−6^	3.61 × 10^−7^	0	1.11 × 10^−7^
D8	1.49 × 10^−5^	1.92 × 10^−5^	2.61 × 10^−5^	1.39 × 10^−4^	3.61 × 10^−6^	1.80 × 10^−6^	3.58 × 10^−8^	2.02 × 10^−8^	9.06 × 10^−8^
D9	2.88 × 10^−5^	2.31 × 10^−5^	7.58 × 10^−5^	1.41 × 10^−4^	3.66 × 10^−6^	0	6.92 × 10^−8^	2.05 × 10^−8^	0
D10	2.41 × 10^−5^	2.23 × 10^−5^	5.97 × 10^−5^	1.54 × 10^−4^	1.02 × 10^−5^	0	5.79 × 10^−8^	2.24 × 10^−8^	0
D11	6.48 × 10^−5^	2.29 × 10^−5^	4.26 × 10^−5^	1.68 × 10^−4^	4.48 × 10^−6^	0	1.56 × 10^−7^	2.43 × 10^−8^	0

**Table 4:** Health risks of typical pollutants in sediment of Danjiangkou Reservoir.

Site	Non-Carcinogenic Risks of Sediment					Carcinogenic Risks of Sediment			
	Cyhalothrin (Lambda)	Fenvalerate I	Dichlorvos	Azinphos-Methyl	Dieldrin	Benz[a]Anth-Racene	Benzo[k]flu-Oranthene	Indeno[1,2,3-cd]Pyrene	β-HCH
R1	3.02 × 10^−5^	1.79 × 10^−5^	2.82 × 10^−5^	1.37 × 10^−4^	1.44 × 10^−5^	1.38 × 10^−11^	1.70 × 10^−12^	3.65 × 10^−10^	1.36 × 10^−9^
R2	1.67 × 10^−4^	5.74 × 10^−5^	1.55 × 10^−4^	0	2.12 × 10^−5^	1.37 × 10^−11^	2.27 × 10^−11^	0	0
R3	2.66 × 10^−5^	3.39 × 10^−5^	4.92 × 10^−5^	1.44 × 10^−4^	3.06 × 10^−6^	1.79 × 10^−11^	1.36 × 10^−12^	3.72 × 10^−10^	1.24 × 10^−9^
R4	3.27 × 10^−5^	1.86 × 10^−5^	2.67 × 10^−5^	1.56 × 10^−4^	1.88 × 10^−6^	1.07 × 10^−11^	0	3.79 × 10^−10^	1.17 × 10^−9^
R5	2.66 × 10^−5^	1.62 × 10^−5^	2.53 × 10^−5^	1.50 × 10^−4^	0	1.33 × 10^−11^	3.49 × 10^−12^	0	0
H1	9.64 × 10^−5^	4.18 × 10^−5^	1.28 × 10^−4^	1.21 × 10^−4^	1.34 × 10^−5^	1.01 × 10^−11^	0	3.63 × 10^−10^	8.16 × 10^−10^
H2	4.40 × 10^−5^	2.01 × 10^−5^	6.04 × 10^−5^	1.40 × 10^−4^	1.31 × 10^−5^	1.17 × 10^−11^	0	0	1.39 × 10^−9^
H3	1.25 × 10^−4^	4.57 × 10^−5^	9.47 × 10^−5^	0	1.47 × 10^−5^	1.48 × 10^−11^	2.57 × 10^−11^	1.18 × 10^−9^	4.73 × 10^−10^
H4	4.40 × 10^−5^	2.01 × 10^−5^	7.30 × 10^−5^	1.43 × 10^−4^	1.30 × 10^−5^	1.54 × 10^−11^	5.78 × 10^−12^	3.55 × 10^−10^	4.66 × 10^−10^
H5	3.13 × 10^−5^	1.89 × 10^−5^	5.31 × 10^−5^	1.25 × 10^−4^	5.71 × 10^−6^	1.03 × 10^−11^	0	3.72 × 10^−10^	1.44 × 10^−9^
H6	4.72 × 10^−5^	2.51 × 10^−5^	6.31 × 10^−5^	1.58 × 10^−4^	9.61 × 10^−6^	1.33 × 10^−11^	4.81 × 10^−13^	3.48 × 10^−10^	7.40 × 10^−10^
H7	4.99 × 10^−5^	2.47 × 10^−5^	3.97 × 10^−5^	1.34 × 10^−4^	1.41 × 10^−5^	1.80 × 10^−11^	4.55 × 10^−11^	6.19 × 10^−10^	0
D1	3.20 × 10^−5^	3.05 × 10^−5^	5.49 × 10^−5^	1.80 × 10^−4^	0	1.19 × 10^−11^	4.32 × 10^−12^	4.26 × 10^−10^	5.45 × 10^−10^
D2	5.24 × 10^−5^	3.60 × 10^−5^	8.13 × 10^−5^	0	1.02 × 10^−5^	1.61 × 10^−11^	0	0	3.41 × 10^−9^
D3	2.82 × 10^−5^	3.76 × 10^−5^	6.92 × 10^−5^	1.54 × 10^−4^	2.24 × 10^−6^	1.47 × 10^−11^	0	3.53 × 10^−10^	4.94 × 10^−10^
D4	4.01 × 10^−5^	2.75 × 10^−5^	3.77 × 10^−5^	1.47 × 10^−4^	2.87 × 10^−6^	4.96 × 10^−12^	4.85 × 10^−13^	0	5.53 × 10^−10^
D5	4.25 × 10^−5^	2.80 × 10^−5^	5.00 × 10^−5^	0	1.07 × 10^−5^	9.47 × 10^−12^	4.02 × 10^−11^	6.08 × 10^−10^	3.53 × 10^−10^
D6	1.49 × 10^−4^	4.72 × 10^−5^	1.26 × 10^−4^	0	1.50 × 10^−5^	1.06 × 10^−11^	0	0	3.69 × 10^−10^
D7	1.51 × 10^−4^	6.26 × 10^−5^	1.45 × 10^−4^	0	2.37 × 10^−5^	1.11 × 10^−11^	3.45 × 10^−12^	4.11 × 10^−10^	8.96 × 10^−10^
D8	1.49 × 10^−5^	1.92 × 10^−5^	2.61 × 10^−5^	1.39 × 10^−4^	3.61 × 10^−6^	1.35 × 10^−11^	5.41 × 10^−12^	4.43 × 10^−10^	1.83 × 10^−9^
D9	2.88 × 10^−5^	2.31 × 10^−5^	7.58 × 10^−5^	1.41 × 10^−4^	3.66 × 10^−6^	1.33 × 10^−11^	0	3.97 × 10^−10^	1.57 × 10^−9^
D10	2.41 × 10^−5^	2.23 × 10^−5^	5.97 × 10^−5^	1.54 × 10^−4^	1.02 × 10^−5^	1.38 × 10^−11^	1.10 × 10^−11^	7.80 × 10^−10^	4.45 × 10^−10^
D11	6.48 × 10^−5^	2.29 × 10^−5^	4.26 × 10^−5^	1.68 × 10^−4^	4.48 × 10^−6^	8.09 × 10^−12^	0	0	9.46 × 10^−10^

## Data Availability

The raw data supporting the conclusions of this article will be made available by the authors on request.

## Supplementary Materials

The following supporting information can be downloaded at: https://www.mdpi.com/article/10.3390/toxics12120859/s1, Table S1: Danjiangkou Reservoir Points Information; Table S2: Recovery, method detection limit (MDL) and method quantification limit (MQL) of pesticides in surface water; Table S3: Recovery, method detection limit (MDL) and method quantification limit (MQL) of pesticides in sediment; Table S4: Aquatic toxicity data and Predicted No Effect Concentration (PNEC) values for polycyclic aromatic hydrocarbons (PAHs) and organophosphorus (OPs) and organochlorine (OC) pesticides in surface water samples; Table S5: Aquatic biotoxicity data for organophosphorus (OP), organochlorine (OC), and pyrethroid pesticides, and polycyclic aromatic hydrocarbons (PAH) in sediment samples, and logarithmic absorption constants (K_oc_) and log K_ow_ for organic compounds; Table S6: Exposure parameters used for estimation of human health risk; Table S7: Reference dose (RfD) and slope factor (SF) for each pollutant; Table S8: Evaluation of non-carcinogenic risks of surface water pollutants; Table S9: Evaluation of carcinogenic risks of surface water pollutants; Table S10: Evaluation of non-carcinogenic risks of sediment pollutants; Table S11: Evaluation of carcinogenic risks of sediment pollutants.
