# Supplementary material for: Distribution of Polycyclic Aromatic Hydrocarbons and Pesticides in Danjiangkou Reservoir and Evaluation of Ecological Risk
Source: Toxics. 2024 Nov 27;12(12):859. doi: 10.3390/toxics12120859 (PMC11680053; doi:10.3390/toxics12120859)
Supplement: Supplementary file 1 [file toxics-12-00859-s001.zip › toxics-3301066-supplementary.pdf]

Article

Distribution of polycyclic aromatic hydrocarbons and pesticides in Danjiangkou Reservoir and evaluation of ecological risk

Ruiwen Li [1#], Hao Pang [3#], Yemin Guo [1], Xuan Zhou [3], Kaiyu Fu [2], Taotao Zhang [4], Jian Han [2], Lihua Yang [2*], Bingsheng Zhou [2], Si Zhou [5*]

[1] Ecology and Environment Monitoring and Scientific Research Center, Ecology and Environment Administration of Yangtze River Basin, Ministry of Ecology and Environment, 430010, Wuhan, China

[2] Key Laboratory of Breeding Biotechnology and Sustainable Aquaculture, Institute of Hydrobiology, Chinese Academy of Sciences, Wuhan 430072, China

[3] School of Chemistry and Environmental Engineering, Wuhan Institute of Technology, Wuhan 430074, China

[4] School of Laboratory Medicine, Hubei University of Chinese Medicine, Wuhan 430065, China

[5] Guizhou Institute of Environmental Science, Guiyang 550081, China

[#] Ruiwen Li and Hao Pang contributed equally as co-first authors.

* Correspondence:

Lihua Yang

Address：Key Laboratory of Breeding Biotechnology and Sustainable Aquaculture, Institute of Hydrobiology, Chinese Academy of Sciences，Wuhan 430072, China

Email：lhyang@ihb.ac.cn

Si Zhou

Address：Guizhou Institute of Environmental Science, Guiyang 550081, China

Email：sparklingenergy@163.com;

Table S1 Danjiangkou Reservoir Points Information

Site	Name	lati-<br>tude	longitude
R1	Han River	32.7981	110.9969
R2	Si River	32.7239	110.9496
R3	Jianhe River	32.5544	111.0695
R4	Guanshan River	32.5286	111.0108
R5	Lang River	32.4311	111.2439
H1	Han Reservoir	32.7394	111.0374
H2	Han Reservoir	32.7282	111.1082
H3	Han Reservoir	32.6577	111.2271
H4	Han Reservoir	32.6060	111.1723
H5	Han Reservoir	32.5293	111.1479
H6	Han Reservoir	32.5728	111.3283
H7	Han Reservoir	32.5609	111.4898
D1	Dan Reservoir	32.5647	111.4826
D2	Dan Reservoir	32.6021	111.5101
D3	Dan Reservoir	32.6533	111.5365
D4	Dan Reservoir	32.7003	111.4922
D5	Dan Reservoir	32.6728	111.6403
D6	Dan Reservoir	32.7314	111.5801
D7	Dan Reservoir	32.7710	111.6325
D8	Dan Reservoir	32.8223	111.5933
D9	Dan Reservoir	32.8657	111.5334
D10	Dan Reservoir	32.9659	111.4122
D11	Dan Reservoir	32.9610	111.5008

Table S2 Recovery, method detection limit (MDL) and method quantification limit (MQL) of pesticides in surface water.

	Surface water		
Analyte	Recoveries (%)	MDLs (ng/L)	MQLs (ng/L)
**PAHs**			
Naphthalene	94.55	0.301	0.994
Acenaphthylene	94.47	0.294	0.969
Acenaphthene	96.26	0.352	1.160
Fluorene	99.29	0.178	0.589
Phenanthrene	83.98	0.307	1.014
Fluoranthene	102.19	0.681	2.248
Pyrene	89.09	0.778	2.568
Benz[a]anthracene	67.02	0.532	1.757
Benzo[a]pyrene	87.25	1.129	3.727
Indeno[1,2,3-cd]pyrene	77.91	23.152	76.400
**OPs**			
O,O,O-triethyl-Phosphorothioate	96.34	0.128	0.424
Dichlorvos	98.12	0.536	1.768
E-Mevinphos	89.35	-	-
Tetraethylpyrophosphate	70.18	1.338	4.415
Naled	132.18	-	-
Phorate	122.13	0.651	2.149
Simazine	93.41	6.500	21.449
Atrazine	84.10	2.808	9.267
**OCs**			
α-HCH	114.61	0.093	0.307
β-HCH	122.19	0.299	0.985
γ-HCH	134.20	0.698	2.302

Table S3 Recovery, method detection limit (MDL) and method quantification limit (MQL) of pesticides in sediment.

Analyte	Surface water		
	Recoveries (%)	MDLs (ng/g)	MQLs (ng/g)
**PAHs**			
Benz[a]anthracene	60.54	0.033	0.110
Benzo[k]fluoranthene	76.26	0.020	0.067
Indeno[1,2,3-cd]pyrene	80.16	0.036	0.119
**Pys**			
Cyhalothrin (Lambda)	112.51	0.010	0.032
Cypermethrin I	140.77	0.167	0.551
Fenvalerate I	120.93	0.018	0.059
**OPs**			
Dichlorvos	70.76	0.010	0.034
E-Mevinphos	76.64	0.014	0.046
Simazine	80.42	0.017	0.055
Malathion	62.79	0.011	0.036
Prothiofos	91.06	0.005	0.015
Sulprofos	111.20	0.008	0.027
Famphur	60.56	0.008	0.027
Azinphos-methyl	101.75	0.208	0.687
**OCs**			
β-HCH	70.54	0.019	0.063
Pentachloronitrobenzene	95.25	0.185	0.610
δ-HCH	60.27	0.009	0.028
Chlordane-trans	86.53	0.002	0.005
Dieldrin	78.48	0.008	0.028

Table S4 Aquatic toxicity data and Predicted No Effect Concentration (PNEC) values for polycyclic aromatic hydrocarbons (PAHs) and organophosphorus (OPs) and organochlorine (OC) pesticides in surface water samples.

Chemicals	CAS No	Fish(96h) LC_{50} (mg/L)	Daphnid(48h) LC_{50} (mg/L)	Green Alage(96h) EC_{50} (mg/L)	AF	$PNEC_{water}$
**PAHs**						
Naphthalene	91-20-3	6.10	5.94	6.91	1000	6.91×10^{3}
Acenaphthylene	208-96-8	2.28	1.55	2.42	1000	2.42×10^{3}
Acenaphthene	83-32-9	1.70	1.03	1.74	1000	1.74×10^{3}
Fluorene	86-73-7	2.11	1.45	2.33	1000	2.33×10^{3}
Phenanthrene	85-01-8	0.23	0.35	1.47	1000	1.47×10^{3}
Fluoranthene	206-44-0	0.39	0.04	0.66	1000	6.60×10^{2}
Pyrene	129-00-0	0.39	0.29	0.66	1000	6.60×10^{2}
Benz[a]anthracene	56-55-3	0.13	0.10	0.29	1000	2.90×10^{2}
Benzo[a]pyrene	50-32-8	0.04	0.04	0.13	1000	1.30×10^{2}
Indeno[1,2,3-cd]pyrene	193-39-5	0.01	0.01	0.05	1000	5.00×10
**OPs**						
O,O,O-triethyl-Phosphorothioate	126-68-1	43.63	26.27	24.96	1000	2.50×10^{4}
Dichlorvos	62-73-7	1.01×10^{2}	1.86×10^{2}	3.13×10^{2}	1000	3.13×10^{5}
E-Mevinphos	7786-34-7	1.51×10^{2}	3.88×10^{2}	2.44×10^{2}	1000	2.44×10^{5}
Tetraethyl pyrophosphate	107-49-3	2.5×10^{3}	1.82×10^{3}	1.19×10^{3}	1000	1.19×10^{5}
Naled	300-76-5	4.28	0.00	1.03×10^{2}	1000	1.03×10^{5}
Phorate	298-02-2	0.00	0.00	3.48	1000	3.48×10^{3}
Simazine	122-34-9	16.00	1.10	0.10	1000	1.00×10^{2}
Atrazine	1912-24-9	4.50	0.00	20.36	1000	2.04×10^{4}
**OC**						
α-HCH	319-84-6	2.24	1.57	2.76	1000	2.76×10^{3}

β-HCH	319-85-7	2.24	1.57	2.76	1000	2.76×10^{3}
γ-HCH	58-89-9	2.24	1.57	2.76	1000	2.76×10^{3}

Table S5 Aquatic biotoxicity data for organophosphorus (OP), organochlorine (OC), and pyrethroid pesticides, and polycyclic aromatic hydrocarbons (PAH) in sediment samples, and logarithmic absorption constants (K_{oc}) and log K_{ow} for organic compounds.

Chemicals	CAS No	Fish(96h) LC_{50} (mg/L)	Daphnid(48h) LC_{50} (mg/L)	Green Alage(96h) EC_{50} (mg/L)	Koc	Log K_{ow}	AF
**PAHs**							
Benz[a]anthracene	56-55-3	0.13	0.10	0.29	9.97×10^{4}	5.78	1000
Benzo[k]fluoranthene	207-08-9	0.04	0.04	0.19	2.01×10^{5}	6.11	1000
Indeno[1,2,3-cd]pyrene	193-39-5	0.01	0.01	0.05	6.52×10^{5}	6.70	1000
**Pys**							
Cyhalothrin (Lambda)	91465-08-6	2.10×10^{-4}	5.00×10^{-5}	0.02	1.51×10^{5}	6.80	1000
Cypermethrin I	67375-30-8	9.00×10^{-4}	1.00×10^{-3}	0.04	1.40×10^{5}	6.60	1000
Fenvalerate I	51630-58-1	4.20×10^{-4}	5.00×10^{-5}	0.02	5.60×10^{4}	6.20	1000
**OPs**							
Dichlorvos	62-73-7	1.01×10^{2}	1.86×10^{2}	3.13×10^{2}	65.98	1.43	1000
E-Mevinphos	7786-34-7	1.51×10^{2}	3.88×10^{2}	2.44×10^{2}	10.83	0.13	1000
Simazine	122-34-9	16.00	1.10	0.10	83.22	2.18	1000
Malathion	121-75-5	9.00	1.80×10^{-3}	28.97	1.02×10^{2}	2.36	1000
Prothiofos	34643-46-4	2.00×10^{-3}	4.20×10^{-4}	0.03	9.32×10^{3}	5.67	1000
Sulprofos	35400-43-2	11.00	7.50×10^{-4}	0.09	7.32×10^{3}	5.48	1000
Famphur	52-85-7	16.37	0.03	10.98	153	2.23	1000
Azinphos-methyl	86-50-0	0.81	1.00×10^{-3}	5.03	3.42×10^{2}	2.75	1000
**OC**							

β-HCH	319-85-7	2.24	1.57	2.76	$3.92×10^3$	3.72	1000
Pentachloronitrobenzene	82-68-8	0.46	0.34	0.82	$7.16×10^3$	4.64	1000
δ-HCH	319-86-8	2.24	1.57	2.76	$3.92×10^3$	3.72	1000
Chlordane-trans	5103-74-2	$7.00×10^{-3}$	$4.00×10^{-2}$	$8.00×10^{-2}$	$2.50×10^5$	6.16	1000
Dieldrin	60-57-1	$3.00×10^{-2}$	0.20	0.18	$5.32×10^3$	5.40	1000

Table S6 Exposure parameters used for estimation of human health risk.

Parameters	Unit	Values of adults	Parameters	Unit	Values of adults
Water ingestion rate (IR_W)	L/d	2	Intestinal absorption ratio (f)	-	1
Exposure frequency (EF)	d/y	365	Sediment ingestion rate (IRs)	mg/d	100
Exposure duration (ED)	y	70	Conversion factor (CF)	kg/mg	10^{-6}
Body weight (BW)	kg	70	Fraction ingested from contaminated source (FI)	-	1
Averaging time (AT)	d	70×365	Skin surface area available for contact (SA)	cm^2/day	5700
Event duration (TE)	h	0.58	Sediment to skin adherence factor (AF)	mg/cm^2	0.07
Lag time for each pollutant in the body	h	1	Absorption factor (ABS)	-	0.13

(τ)					
Skin permeability parameter (k)	cm/h	0.069	Event frequency (FE)	times/d	0.3

Table S7 Reference dose (RfD) and slope factor (SF) for each pollutant.

pollutant	SF (mg/kg·d)	RfD (mg/kg·d)	pollutant	SF (mg/kg·d)	RfD (mg/kg·d)
Naphthalene	1.20×10^{-1}	2.00×10^{-2}	α-HCH	6.3	8.00×10^{-3}
Acenaphthene	-	6.00×10^{-2}	β-HCH	1.8	2.00×10^{-4}
Fluorene	-	4.00×10^{-2}	γ-HCH	1.3	3.00×10^{-4}
Phenanthrene	-	3.00×10^{-2}	Cyhalothrin (Lambda)	-	9.30×10^{-4}
Fluoranthene	-	4.00×10^{-2}	Cypermethrin I	-	5.00×10^{-3}
Pyrene	-	3.00×10^{-2}	Fenvalerate I	-	2.50×10^{-2}
Benz[a]anthracene	7.30×10^{-1}	-	Mevinphos, E-	-	3.00×10^{-4}
Benzo[a]pyrene	7.30	3.00×10^{-4}	Malathion	-	2.00×10^{-2}
Indeno[1,2,3-cd]pyrene	7.30×10^{-1}	-	Azinphos-methyl	-	3.00×10^{-3}
Dichlorvos	2.90×10^{-1}	5.00×10^{-4}	Pentachloronitrobenzene	-	3.00×10^{-3}
Naled	-	2.00×10^{-3}	Chlordane-trans	5.00×10^{-4}	3.50×10^{-1}
Phorate	-	3.50×10^{-2}	Dieldrin	16.00	5.00×10^{-5}
Simazine	1.20×10^{-1}	5.00×10^{-3}	Benzo[k]fluoranthene	7.30×10^{-3}	-
Atrazine	-	3.50×10^{-2}			

Table S8 Evaluation of non-carcinogenic risks of surface water pollutants.

Site	Acenaphthene	Fluoranthene	Pyrene	Benzo[a]pyrene	Naled	Phorate	Simazine	β-HCH	γ-HCH
R1	0	0	**0**	0	0	0	0	0	0
R2	0	0	0	0	0	0	0	0	1.06×10^{-4}
R3	0	0	0	0	0	0	0	1.61×10^{-4}	0
R4	0	0	1.15×10^{-5}	0	0	0	0	0	0
R5	0	0	0	0	0	0	0	0	0
H1	0	0	0	0	0	0	0	1.51×10^{-4}	0
H2	0	0	0	0	0	0	0	0	0
H3	0	0	0	0	0	0	0	1.48×10^{-4}	0
H4	0	0	0	6.32×10^{-3}	0	0	0	0	1.07×10^{-4}
H5	0	0	0	0	0	0	0	0	0
H6	0	0	0	1.13×10^{-3}	0	0	0	0	0
H7	0	0	0	0	5.72×10^{-4}	1.95×10^{-6}	0	0	0
D1	0	0	0	0	0	0	0	0	0
D2	0	2.66×10^{-6}	0	0	0	0	0	0	0
D3	0	0	0	0	0	0	0	0	0
D4	0	0	0	0	0	0	0	0	0
D5	0	0	0.00	0	0	0	0	0	0
D6	5.24×10^{-8}	0	0.00	0	0	0	0	0	0
D7	0	0	0	0	0	0	0	0	1.04×10^{-4}
D8	0	0	0	0	0	0	0	1.62×10^{-4}	0
D9	0	0	0	7.16×10^{-4}	0	0	0	0	0
D10	0	5.40×10^{-6}	0	0	0	0	0	0	0
D11	0	0	0	0	0	0	0	0	0

Table S9 Evaluation of carcinogenic risks of surface water pollutants.

Site	Benz[a]anthracene	Benzo[a]pyrene	Indeno[1,2,3-cd]pyrene	Simazine	β-HCH	γ-HCH
R1	0	0	0	0	0	0
R2	0	0	0	0	0	4.13×10^{-8}
R3	0	0	1.57×10^{-5}	0	5.79×10^{-8}	0
R4	0	0	0	0	0	0
R5	0	0	1.01×10^{-6}	0	0	0
H1	0	0	5.96×10^{-6}	0	5.44×10^{-8}	0
H2	0	0	0	0	0	0
H3	0	0	0	0	5.32×10^{-8}	0
H4	2.74×10^{-7}	1.39×10^{-5}	0	0	0	4.16×10^{-8}
H5	0	0	0	0	0	0
H6	0	2.48×10^{-6}	0	0	0	0
H7	0	0	0	0	0	0
D1	0	0	0	0	0	0
D2	0	0	0	0	0	0
D3	0	0	0	0	0	0
D4	0	0	5.06×10^{-6}	0	0	0
D5	0	0	0	0	0	0
D6	0	0	0	0	0	0
D7	0	0	0	0	0	4.04×10^{-8}
D8	0	0	0	0	5.82×10^{-8}	0
D9	0	1.57×10^{-6}	0	0	0	0
D10	0	0	0	0	0	0
D11	0	0	0	0	0	0

Table S10 Evaluation of non-carcinogenic risks of sediment pollutants.

Site	Cypermethrin I	E-Mevinphos	Simazine	Malathion	β-HCH	Pentachloronitrobenzene	Chlordane-trans
R1	0	0	**0**	0	0	0	0
R2	0	0	0	0	0	0	0
R3	0	0	0	0	0	0	0
R4	0	0	1.15×10^{-5}	0	0	0	0
R5	0	0	0	0	0	0	0
H1	0	0	0	0	0	0	0
H2	0	0	0	0	0	0	0
H3	0	0	0	0	0	0	0
H4	0	0	0	6.32×10^{-3}	0	0	0
H5	0	0	0	0	0	0	0
H6	0	0	0	1.13×10^{-3}	0	0	0
H7	0	0	0	0	5.72×10^{-4}	1.95×10^{-6}	0

0	0	0	0	0	0	0
0	2.66×10^{-6}	0	0	0	0	0
0	0	0	0	0	0	0
0	0	0	0	0	0	0
0	0	0	0	0	0	0
5.24×10^{-8}	0	0	0	0	0	0
0	0	0	0	0	0	0
0	0	0	0	0	0	0
0	0	0	7.16×10^{-4}	0	0	0
0	5.40×10^{-6}	0	0	0	0	0
0	0	0	0	0	0	0

Table S11 Evaluation of carcinogenic risks of sediment pollutants.

Site	Dichlor-vos	Simazine	Chlordane-trans	Dieldrin
R1	0	0	1.73×10^{-13}	0
R2	0	0	0	0
R3	8.77×10^{-11}	0	0	0
R4	0	0	7.46×10^{-14}	0
R5	0	5.81×10^{-10}	0	3.41×10^{-8}
H1	8.40×10^{-11}	0	0	0
H2	0	0	0	0
H3	0	0	0	0
H4	0	0	0	0
H5	0	0	0	0
H6	0	0	0	0
H7	0	0	0	0
D1	0	0	0	0
D2	5.33×10^{-10}	0	0	0
D3	0	0	0	0
D4	0	0	0	0
D5	0	0	8.58×10^{-14}	0
D6	7.86×10^{-11}	0	0	0
D7	0	0	0	0
D8	0	1.37×10^{-10}	0	0
D9	0	0	0	0
D10	0	0	0	0
D11	0	0	0	0
